# Pathologic Peri-Implant Proximal Femur Fracture: Takeaways from Our Experience

**DOI:** 10.1155/2023/3193937

**Published:** 2023-11-14

**Authors:** Mustafa Hashimi, Jason A. Shah, Henry M. Gass 4th, Alexander R. Webb, John M. Kopriva, Shervin V. Oskouei

**Affiliations:** ^1^The University of Iowa Carver College of Medicine, Iowa City, IA 52242, USA; ^2^Department of Orthopaedic Surgery, Emory University School of Medicine, Atlanta, GA 30329, USA

## Abstract

Pathologic fractures of the distal femur secondary to bone metastases are not as common as those in the proximal femur, and they are rarely reported on in the literature. Even in the absence of current metastatic lesions in the femoral neck, traditional orthopaedic teaching has stressed the importance of protecting the entire femur, while recent studies have shown that it may not be necessary to stabilize the entire femur in the event of future metastases. Thus, there is no consensus regarding optimal surgical treatment, making the choice of fixation often based on the experience of the surgeon. In this paper, we reported on a patient who presented with a pathologic fracture of the distal femur who was stabilized with a retrograde intramedullary nail and then subsequently suffered a pathologic fracture of the proximal femur. To our knowledge, there have been no cases reported on a peri-implant pathologic fracture proximal to a retrograde intramedullary nail in the setting of metastatic bone disease. We would like to share our experience on how to surgically manage this and discuss the literature around management of distal femoral bone metastases.

## 1. Introduction

Pathologic fractures of the femur are debilitating complications that can occur in patients with metastatic bone disease, primary malignant or benign lesions, or underlying metabolic abnormalities. The femur is the most common long bone affected by bone metastases, with the majority of metastases located proximally [1]. While approximately 10% of all femoral metastases are located distally, these lesions prove difficult to treat with no consensus on the optimal surgical treatment [2].

Even in the absence of current metastatic lesions in the femoral neck, traditional orthopaedic teaching has stressed the importance of protecting the entire femur. Thus, patients may be treated with a cephalomedullary nail to protect the femoral neck in case of potential metastatic development [3]. However, development of future femoral neck metastases and subsequent pathologic fractures are rare, and recent literature has begun to investigate whether prophylactic femoral neck stabilization is necessary in such patients. This supports the rational of using only intramedullary nailing to stabilize the femoral diaphysis primarily [4].

The patient described in this report presented with a pathologic fracture of the distal femur who was stabilized with a retrograde intramedullary nail and then subsequently suffered a pathologic fracture of the proximal femur. One case series has been reported on low energy peritrochanteric fractures in the presence of a retrograde nail [5]. However, to our knowledge, there has been no cases reported on a peri-implant fracture proximal to a retrograde intramedullary nail in the setting of metastatic bone disease. We would like to share our experience on how to surgically manage this and discuss the literature around management of distal femoral bone metastases. A consent to publish was retrospectively obtained from the patient of interest prior to publication of this case report.

## 2. Case Report

**Figure 1:**
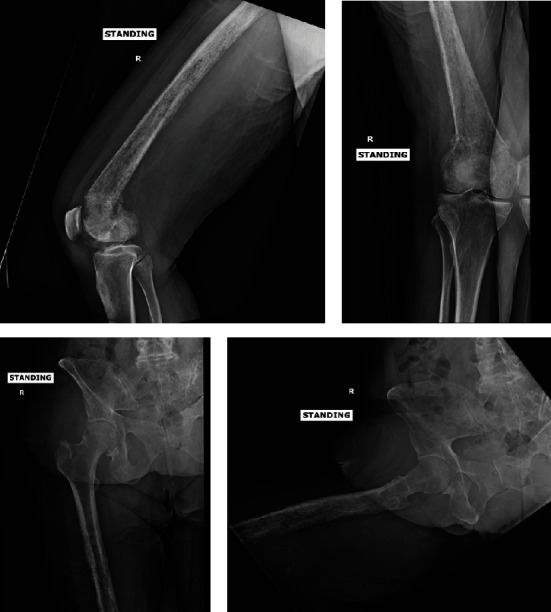
AP and lateral radiograph views of the femur revealing a healing, slightly dorsally angulated pathologic distal femur fracture with diffuse changes throughout the entire diaphysis.

A 62-year-old female was referred to our institution for a right distal femur fracture with radiographs concerning for an underlying neoplastic process. She initially developed right knee pain after attempting to stand from a chair. At that time, she was seen by an outside provider at a local urgent care where no imaging was obtained. She had no obvious deformity and was able to ambulate with an assistive device and was discharged home with a Medrol dose pack. She continued to have severe, persistent pain with limited range of motion a month later and was evaluated by a local orthopedist. Radiographs of the femur identified a healing, slightly dorsally angulated distal femur fracture with diffuse changes throughout the entire diaphysis (Figure 1).

**Figure 2:**
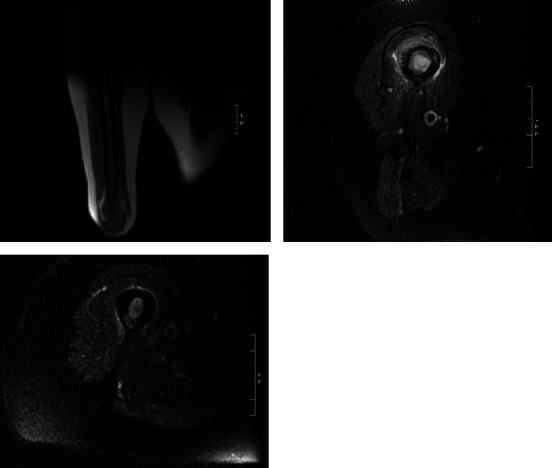
Coronal and axial magnetic resonance imaging of femur demonstrating metastatic bone disease.

Magnetic resonance imaging (MRI) was obtained, and she was subsequently referred to an orthopaedic oncologist at our institution and was seen in clinic one month later. Further questioning revealed a family history of breast cancer but stated that she had a negative mammogram the prior year. She denied antecedent right hip pain prior to developing right knee pain, or pain in any other extremities. Further review of radiographs revealed diffuse lytic changes surrounding the fracture and extending throughout the diaphysis without femoral neck involvement. Review of the magnetic resonance imaging (MRI) that was obtained prior did not show soft tissue involvement (Figure 2).

**Figure 3:**
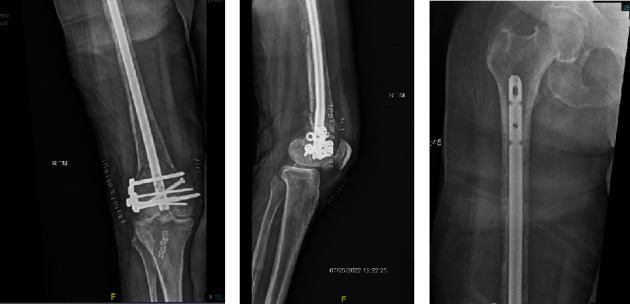
Postoperative radiographs of the femur showing properly placed retrograde femoral nailing system.

There was no known primary tumor, so primary bone tumor or metastatic disease was thought to be the etiology of her pathologic fracture. We planned for an open biopsy with frozen analysis and retrograde intramedullary nail with lateral locking washer. Pathologic frozen analysis of the initial opening marrow reamings revealed features consistent with metastatic breast carcinoma, so we proceeded with retrograde intramedullary fixation (Figure 3). Her immediate postoperative course was unremarkable, and she was able to ambulate with a walker on postoperative day one. She was discharged home the next day with plan to follow up with oncology.

**Figure 4:**
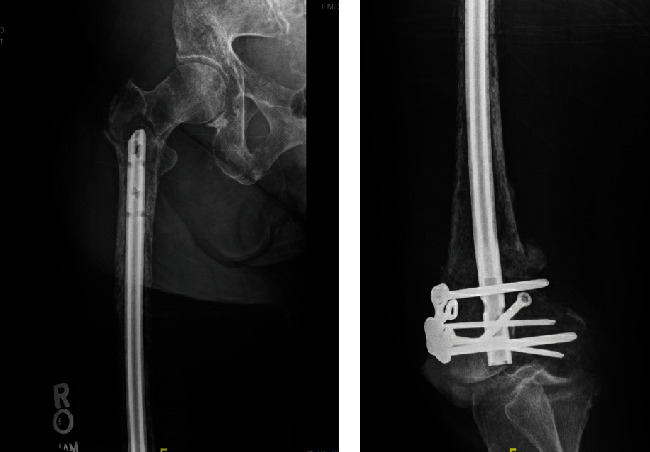
Radiograph of femur revealing right pathologic peri-implant intertrochanteric femur fracture.

**Figure 5:**
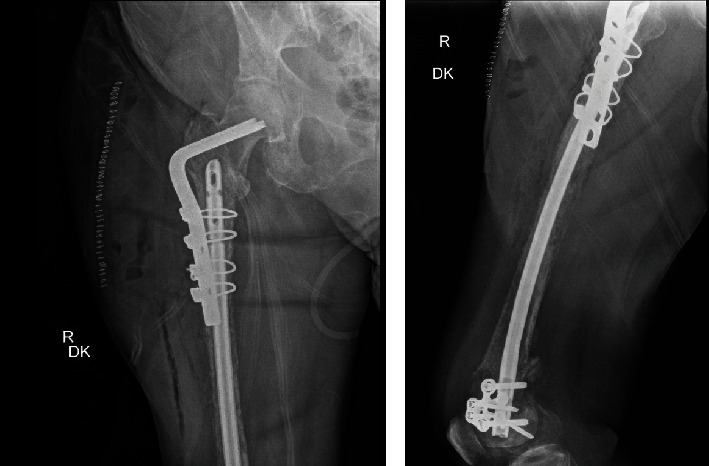
Postoperative radiographs of femur demonstrating fracture fixation with 95-degree angled blade plate and cerclage wires.

The patient returned to the emergency department 20 days postoperatively with right hip pain after sustaining a fall in her kitchen. Radiographs revealed an intertrochanteric femur fracture just proximal to the intramedullary nail (Figure 4). She successfully underwent open reduction and internal fixation (ORIF) with a 95-degree angled blade plate given the location of the fracture and the limited space above the previous retrograde nail (Figure 5). Tissue samples taken at that time confirmed presence of metastatic disease. On postoperative day one, she was transfused 2 units of PRBC for postoperative anemia. CT of the chest/abdomen/pelvis was also obtained during her hospital stay which revealed a large left breast mass with enlarged lymph nodes, multiple lung nodules, and likely bone metastases to the scapula, sternum, left proximal humerus, and ribs. On postoperative day four, she was discharged home weight-bearing as tolerated with a walker.

**Figure 6:**
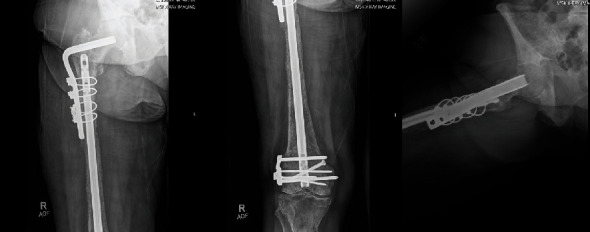
Two-month postoperative radiographs demonstrating healing of previous pathologic peri-implant femur fracture without concern for hardware failure.

The patient returned to clinic 6 months postoperative where she was ambulating with a cane for assist and imaging demonstrating healing of her pathologic proximal femur fracture without concern for hardware failure (Figure 6). Radiotherapy was considered postoperatively for pain control in conversation with our radiation oncologist. A joint decision was made to forego radiation treatment till this point in time and pursue hormone-targeted therapy.

## 3. Discussion

Pathologic fractures of the distal femur secondary to bone metastases are not as common as those in the proximal femur, and they are rarely reported on in the literature. According to a large retrospective database study done by Willeumier et al., approximately 10% of all femoral metastases were located distally [2]. While there are many potential surgical treatments including endoprosthetic reconstruction, plate fixation, intramedullary nail fixation, and arthroplasty, the choice of surgical fixation is often based on clinical experience given the scarcity of literature regarding treatment of distal femoral pathologic fractures [6–9]. In a systematic review by Willeumier et al., only two studies were found that reported outcomes specific to this fracture type [6].

Traditional orthopaedic teaching has stressed the importance of protecting the entire femur in the setting of metastatic bone disease, which provides the basis to use cephalomedullary implants. A study by van der Hulst et al. included 31 patients with impending or actual pathologic fractures of the femur that were treated with intramedullary nailing. Five femoral neck fractures occurred within 5 months of the femoral nailings. From these results, they concluded that the entire femur should be stabilized to prevent the occurrence of a second fracture and need for reoperation [3].

While the rationale behind protecting as much femur as possible is sensible, there are very few studies in the literature that provide support for this practice [3, 9]. Investigators have recently began to study whether it is necessary to stabilize the entire femur in the event of future metastases. A study by Moon et al. looked at 141 patients with isolated femoral diaphyseal lesions who were treated with cephalomedullary nailing and found that no patients in this series developed femoral neck metastases postoperatively. These findings are in stark contrast to the findings by van der Hulst et al. The authors concluded that their results did not support the use of cephalomedullary implants for the sole purpose of prophylactic femoral nail stabilization [4]. A study by Boden et al. reviewed 68 patients with impending or actual pathologic fractures of the proximal femur who then underwent surgical stabilization. They found only 1 patient who developed new distal femoral metastasis and 3 patients with local progression of disease, none of which who required further intervention [10].

A study by Alvi and Damron reviewed 96 patients who had undergone surgical stabilization of the femur or humerus using intramedullary fixation or long-stem arthroplasty in the setting of metastases, myeloma, or lymphoma. While 11 patients experienced local bony disease progression, only 1 patient developed a new discrete lesion in the bone. More notable, however, was that 12 patients had physiologic complications that were potentially attributed to the long intramedullary implant, including two patients who had a pulmonary embolism in the postoperative period [11].

These studies suggest that there is a low likelihood of developing metastases in the femur after treated isolated lesions. While stabilizing the entire femur proves to be beneficial in such patients who do develop discrete lesions, it seems that the risk of physiologic complications increases with the use of long intramedullary implants [10]. Specifically, a long cephalomedullary implant does come with the risk of joint penetration, increased blood loss and operative time, as well as increased radiation exposure when compared to the standard retrograde nail [4].

The supporting literature, as well as the fact that the patient in our case did not have evidence of femoral neck metastasis, supported our rationale in using a retrograde intramedullary nail without additional femoral neck protection. Unfortunately, our patient subsequently suffered a pathologic fracture just proximal to the implant only 20 days postoperatively. In the study by van Der Hulst et al., all reported femoral neck fractures occurred within 5 months of the initial femoral nailings. Given the short time frame, they concluded that the fractures likely occurred as a result of metastatic disease already present before surgery [3]. Although there was no evidence of lesions in the femoral neck upon initial radiographs in our case, tissue diagnosis at time of the second procedure confirmed the presence of metastatic disease. At the time of surgery, MRI was obtained at an outside institution and did not visualize the femoral neck. In retrospect, thorough evaluation of the femoral neck with MRI would have demonstrated evidence of metastasis and ultimately prevented this complication.

In simple cases with a stable nonpathologic intertrochanteric fracture, a sliding hip screw (SHS) is often considered a treatment of choice [12]. Our initial thought for fixation of the peri-implant fracture was the SHS; however, review of radiographs demonstrated that there was limited space above the proximal end of the retrograde nail to put in the SHS. Mounasamy et al. reported on two cases of femoral fractures at the proximal aspect of a retrograde nail. Both cases were treated with removal of the hardware followed by cephalomedullary antegrade nailing [13]. This option was not possible in our case given that the previously treated distal pathologic fracture was not yet completely healed. In addition, removal of the retrograde nail does come with increased operating time as well as the risk of damaging the articular cartilage in the knee. In addition, curettage and cementation was considered but deemed nonfeasible due to the extent of metastatic disease and cortical disruption.

Our choice of implant for fixation of the peri-implant fracture was the 95-degree angled blade plate given the fracture pattern and available space above the retrograde nail. This implant has been shown to be an excellent surgical treatment to restore anatomical alignment and maintain bony union in certain proximal femur fracture patterns [14, 15]. Upon reviewing of the literature following our case, we found very few cases that reported on this fracture pattern and none in the setting of metastatic bone disease. The largest case series was one done by Yvette et al. that reported on 7 patients who suffered low energy peritrochanteric fractures at the proximal aspect of a retrograde nail. Three of the patients were treated with a SHS, three patients were treated with a 95-degree extramedullary device, and one patient was treated with removal of retrograde nail and insertion of a long cephalomedullary nail. All fractures healed with no implant failure or major complications. The authors discussed a treatment algorithm that was dependent on fracture type, available space above the retrograde nail, and the condition of the previous distal fracture [5].

The current available literature, as well as our experience in this case, proves that the treatment of pathologic fractures is difficult, especially in the distal femur. While the studies reported in this case study demonstrate the low likelihood of development of discrete metastatic lesions, more research with larger patient sample sizes must be performed before supporting a clinical practice that favors entire femur stabilization versus local disease stabilization. This case demonstrates a rare but potential outcome that can occur in such patients with distal femur metastasis and a method to manage these complex fractures. Careful attention to evaluating the extent of bony involvement of metastatic disease as well as implant choice and construct design is paramount in the surgical management of these patients.

## Data Availability

The radiographic and clinical data used to support the findings of this study are included within the article.
